# Effects of Current-injection Firing with Ag Paste in a Boron Emitter

**DOI:** 10.1038/srep21553

**Published:** 2016-02-10

**Authors:** Chanseok Kim, Jae-Wook Choi, Sungjin Choi, Soomin Kim, Hyomin Park, Hee-eun Song, Sam S. Yoon, Joo-Youl Huh, Yoonmook Kang, Hae-Seok Lee, Donghwan Kim

**Affiliations:** 1 Department of Materials Science and Engineering, Korea University, Seoul 136-713, Republic of Korea; 2 Photovoltaic Laboratory, Korea Institute of Energy Research, Daejeon 305-343, Republic of Korea; 3 School of Mechanical Engineering, Korea University, Seoul 136-713, Republic of Korea; 4 Korea University-Korea Institute of Science and Technology Green School, School of Energy and Environment, Korea University, Seoul 136-713, Republic of Korea

## Abstract

A high contact resistance for screen-printed contacts was observed when a conventional Ag paste was used on a boron emitter. The results of this study suggest that electron injection during firing is one of the processes that contribute to a lower contact resistance. Larger quantities of Ag precipitates formed upon electron injection into the boron emitter, which was confirmed by observing Ag crystallite or dendrite structures on the boron and by measuring the contact resistance between the boron emitter and the Ag bulk. The electron-injected sample had approximately 10000 times lower contact resistance than an untreated sample. The contact resistance of the electron-injected sample was 0.021 mΩ∙cm^2^ under optimal conditions, which is lower than that of conventional p-type silicon solar cells. Thus, electron injection can effectively lower contact resistance when using Ag paste in n-type silicon solar cells. During the cooling in the firing process, dissolved Ag ions in the glass layer are formed as dendrites or crystallites/particles. The dendrites are formed earlier than others via electrochemical migration under electron injection conditions. Then, crystallites and particles are formed via a silicon etching reaction. Thus, Ag ions that are not formed as dendrites will form as crystallites or particles.

N-type silicon wafers are particularly advantageous for the design of high-efficiency solar cells because metal impurities are less detrimental to n-type silicon, which leads to a higher minority carrier lifetime^1^. Additionally, n-type silicon solar cells do not undergo light-induced degradation, which reduces cell performance^2^. However, contact problems arise for boron emitters that are applied to n-type silicon solar cells using the screen-printing technique. After firing with Ag paste, the contact resistance is higher than a phosphorus emitter; this problem can be resolved by adding aluminum to the Ag paste^3^,^4^,^5^,^6^. However, this produces other problems, such as shunting behavior and a high line resistance^3^. The Ag becomes less dense as the aluminum content increases^6^; thus, pastes with little or no aluminum are beneficial. In a recent study, Engelhardt *et al.* reported a contact resistivity of approximately 1 mΩ∙cm^2^ with a boron emitter using Ag paste^7^ when B-doped SiO_x_ (SiO_x_:B) was used as the passivation layer. They reported a low contact resistivity at a peak temperature of 850 °C even above the Ag-Si eutectic point. However with a peak wafer temperature higher than the Ag-Si eutectic point, many Ag crystallites embed into the n-type base that causes a shunt path and recombination. When phosphorus emitters are fired with Ag paste, the Ag nucleates on the silicon emitter surface and grows as a crystallite. In a recent study by Hong, K. *et al.*, Ag (Ag^+^) and oxygen (O^2−^) ions etched a phosphorus emitter, which was found to contribute to Ag crystallite formation during the firing process^8^. Also, Schubert reported that lead oxide (PbO) etches the silicon emitter^9^. Both of these reports confirm that the silicon etching reaction is critical for Ag crystallite formation. However this effect would be markedly reduced in boron emitters. In solution etching, silicon is etched in an alkaline solution such as a potassium hydroxide (KOH) solution. The silicon surface is etched with hydroxide ions (OH^−^) that are generated by the decomposition of water molecules (H_2_O) using the electrons at the silicon surface; thus, electrons are important in the etching of silicon. The etching reaction is particularly weak in emitters with high levels of boron doping due to the presence of fewer electrons. The etch rate is markedly reduced at boron concentrations above 10^19^ cm^−3^ in alkaline solutions^10^. Similar to the etching process in alkaline solutions, electrons play a significant role in the etching of silicon during the paste etching process. Ag ions that are created from the Ag bulk participate in the silicon etching reaction^8^, and electrons that are present at the surface of a boron emitter participate in the Ag ion reduction reaction. Positively charged Ag ions etch the silicon less strongly in boron emitters than in phosphorus emitters.

This study investigated the use of conventional Ag paste in n-type silicon solar cells. We injected electrons into the boron emitter to increase the number of electrons at the boron emitter surface during the firing process. The nano- or micro-structure of Ag and its contact behavior were investigated using field emission scanning electron microscopy (FE-SEM) and the transfer length method.

## Methods

**Figure 1:**
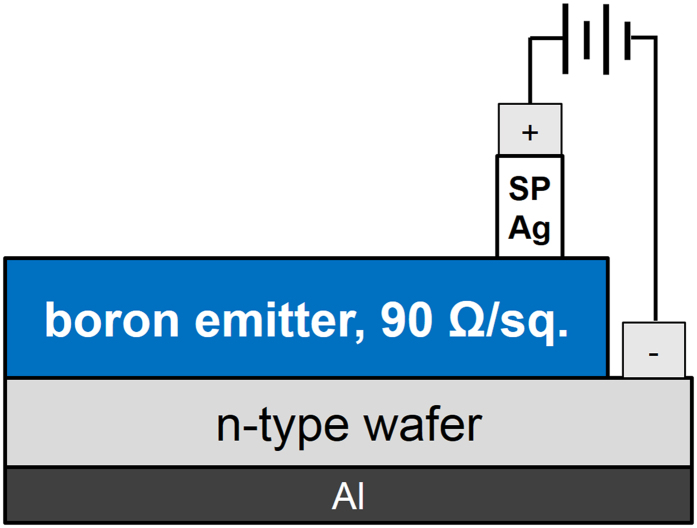
Schematic of the sample structure, which was designed for current injection into the boron emitter.

**Figure 2:**
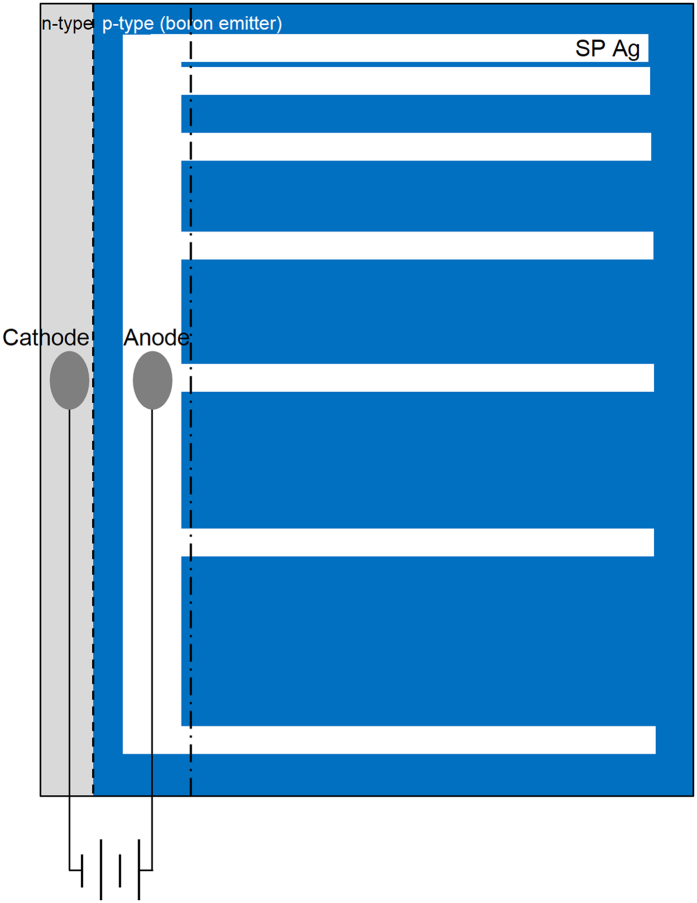
Schematic design of the contact structure that was used for the transfer length method.

Pseudo-square, 6-inch-wide, n-type Czochralski-grown silicon wafers were used in this study. Their resistivities and thicknesses were 1–3 Ω∙cm and 180 μm, respectively. The surfaces of the wafers were textured using a potassium hydroxide (KOH) solution. The textured wafers were cleaned with a hydrochloric acid/hydrogen peroxide mixture (HPM, HCl:H_2_O_2_:H_2_O, 1:1:2) at 80 °C for 10 min. Then, a boron emitter was formed from BBr_3_ and O_2_ in a tube furnace at 940 °C. The tube furnace had a diameter of 306 mm and a length of 1115 mm. The temperature was increased from 800 °C to 940 °C over 30 min, and the sample was maintained in a N_2_ atmosphere for 20 min. The sheet resistance over the entire wafer was kept uniform by controlling the composition of the doping gases, which were BBr_3_, O_2_, and N_2_, in the pre-deposition step via their flow rates of 0.3, 0.06, and 14 L/min, respectively. During the pre-deposition step, the temperature was maintained at 940 °C for 20 min, and a drive-in step was performed under a N_2_ atmosphere at the same temperature for 30 min. The diffused samples were dipped in diluted hydrofluoric acid (HF, 5%) for 5 min to remove the borosilicate glass. The boron-rich layer was then etched for 30 s in an HF/nitric/acetic acid solution that had a ratio of 1:100:25. The mixture was prepared from an aqueous solution; thus, the exact ratio of the mixture was 1:100:25:40 (HF/nitric/acetic acid/water). The edge of the boron emitter was etched using the acid mixture for 5 min. A conventional Ag paste (8600A) was used with the screen-printing method for front metallization, and an aluminum paste was used for the back metal, as shown in Fig. 1. To pattern the front metal to measure the contact resistance, the contact structure was designed as shown in Fig. 2. The exposed n-type wafer was connected to the cathode, and part of the busbar was connected to the anode with Ag wires. The wires were attached to the wafer with Ag conductive ink. The distances between the fingers were 0.5, 1, 1.5, 2, 2.5, and 3 mm, respectively. The drying and firing processes were conducted in a rapid thermal annealing (RTA) chamber. All of the samples were dried for 1 min at 473 K with N_2_ gas. After drying, the Ag wires connected the screen-printed Ag to the boron emitter and exposed the n-type wafer. The wires were connected to a Keithley source-measurement unit (Model 238, Keithley Instruments Inc., USA) for current injection. Starting with the firing process, electrons were injected into the boron emitter using the Ag bulk as the anode and the n-type wafer as the cathode. We varied the amount of injected current density from 0 to 5 A/cm^2^; the peak wafer temperature was just below the Ag-Si eutectic temperature. We fabricated the samples three times and measured three times to determine the reproducibility and uncertainty. We also increased the cooling rate via water quenching. The firing processes were performed under various ambient gases. After the firing process, the Ag wires were removed from the samples. The busbar and exposed n-type wafer region were cut along the dashed-dotted line, as shown in Fig. 2. The remaining sample, which consisted only of finger lines, was used to measure the contact resistance via the transfer length method. The finger width after firing was 90 μm. The contact resistance of each sample was calculated from the current-voltage (I-V) characteristics that were measured using a source meter via the transfer length method. The Ag bulk and glass layer were etched before the nano- or micro-structures were observed. Diluted nitric acid and diluted HF were used as the Ag bulk etchant and glass-layer etchant, respectively. The front nano- or micro-structures were observed using an FE-SEM (Quanta 250 FEG, FEI, USA) at an accelerating voltage of 15 kV.

## Results

### Structure for electron injection into the boron emitter

We fabricated a sample structure for electron injection into the boron emitter, as shown in Fig. 1. A boron emitter that was formed of borosilicate glass was generated on an n-type textured wafer in a thermal furnace. Its edge was etched with the acid mixture, and Ag paste was deposited onto the boron emitter while aluminum was deposited onto the opposite side. The aluminum forms an Al-doped layer on the rear side after the firing process but does not influence the contact formation on the front; this simply blocks the light of the lamp in the RTA chamber. After drying, Ag wires were connected to the Ag bulk and to the n-type silicon surface. We selected the Ag bulk as the anode and the n-type silicon as the cathode for the firing process. The electrons were injected into the boron emitter through the n-type silicon surface, and the two conductors were separated with a glass layer. The Ag ions dissolved in the glass layer and migrated to the boron emitter through the glass layer. On the boron emitter surface, the electrodeposited Ag grew in the form of dendrites or particles/crystallites, which formed an electrical connection between the boron emitter and the Ag bulk. The dendrites had a tree-like structure of crystals, crystallites/particles and rounded- or elongated-shaped crystals. The crystallites were embedded in the silicon, and particles floated in the glass layer or formed on the silicon.

### Effect of electron injection into the boron emitter

**Figure 3:**
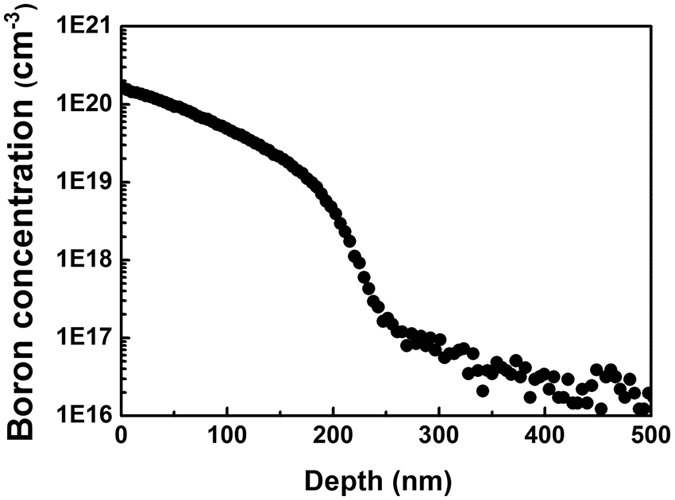
Doping profile of the boron emitter that was used in this study.

**Figure 4:**
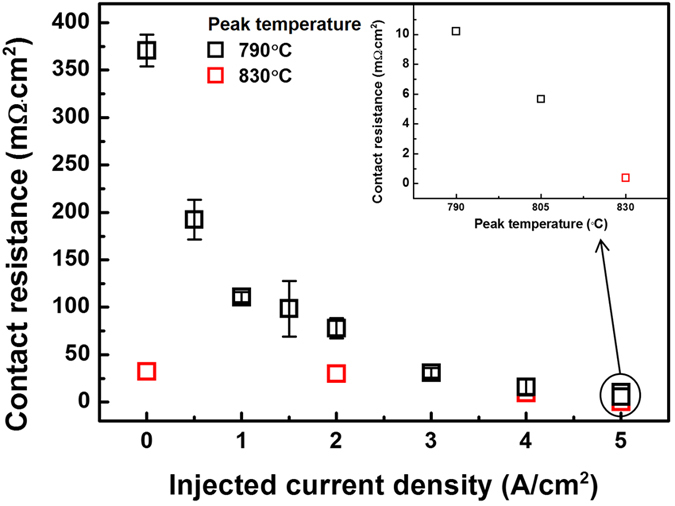
Contact resistance between the Ag bulk and boron emitter versus the injected current density. In the inset, the variation in the contact resistance with the peak wafer temperature is shown for an injected current density of 5 A/cm^2^.

The boron emitter profile used in this study is shown in Fig. 3, which was measured via secondary ion mass spectroscopy (SIMS). The maximum dopant concentration at the surface was 1.9E20 cm^−3^. The junction depth was approximately 300 nm. To investigate the contact resistance between the boron emitter and the Ag bulk using the transfer length method, we measured the I–V characteristics of the samples. Figure 4 shows the contact resistance versus the injected current density. Table 1 shows the specific values of the contact resistance versus the injected current density at a 790 °C peak wafer temperature. A higher current density led to lower contact resistance, which suggests that larger amounts of electrons that are injected into the boron emitter yield a lower contact resistance when using the screen-printing technique. The lowest contact resistance of the sample was 0.021 mΩ∙cm^2^, which indicates a nearly lossless resistance in the metal/silicon contact in a conventional solar cell structure. Table 2 shows the contact resistance versus the injected current density at a 830 °C peak wafer temperature. The contact resistances are much lower than 790  °C peak wafer temperature, and the gap narrows at a high current density. The contact resistance at 5 A/cm^2^ was 0.373 mΩ∙cm^2^, which is below 1 mΩ∙cm^2^.

**Figure 5:**
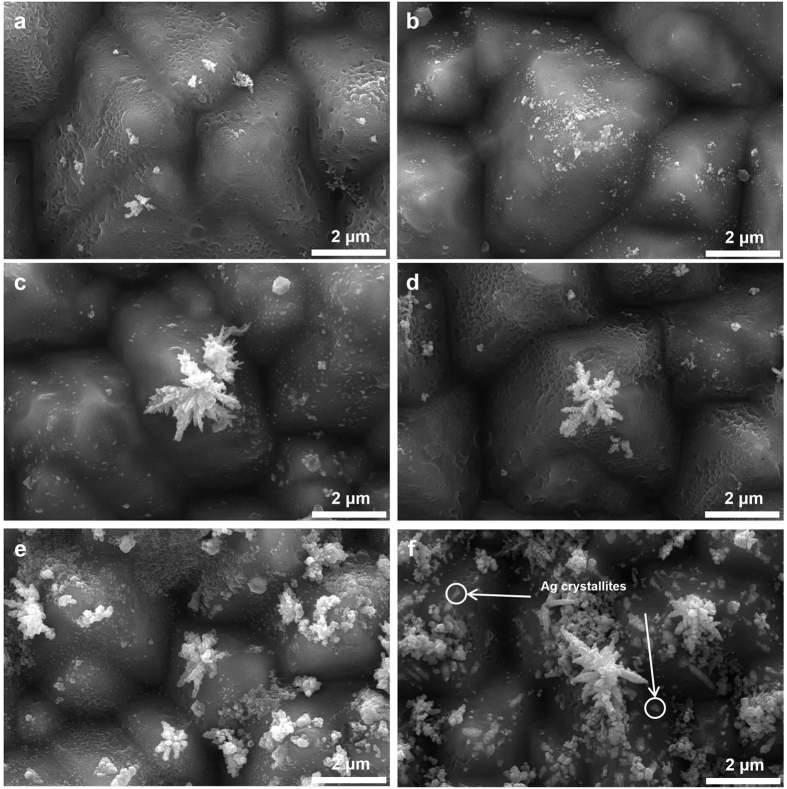
Plane-view SEM images of the Ag precipitates that were fabricated with the injected current densities of (**a**) 0 A/cm^2^, (**b**) 1 A/cm^2^, (**c**) 2 A/cm^2^, (**d**) 4 A/cm^2^, and (**e**) 5 A/cm^2^ and (**f**) under optimal conditions.

Ag formation on the surface of the boron emitter was observed using FE-SEM. The injecting current density controlled the number of electrons at the surface of the boron emitter. The Ag bulk and glass layer were etched to closely examine the Ag precipitates. Figure 5a–f present plane-view images at different injected current densities. For the injected current densities from 0 to 1 A/cm^2^ (Fig. 5a–b), nano-sized Ag particles were deposited onto the boron emitter, which grew into a dendritic structure, as shown in Fig. 5c–f. This structure enhanced the electrical conduction from the boron emitter to the Ag bulk. Under optimum conditions (Fig. 5f), Ag crystallites and dendrites were observed and led to a markedly lower contact resistance between the boron emitter and Ag bulk.

**Figure 6:**
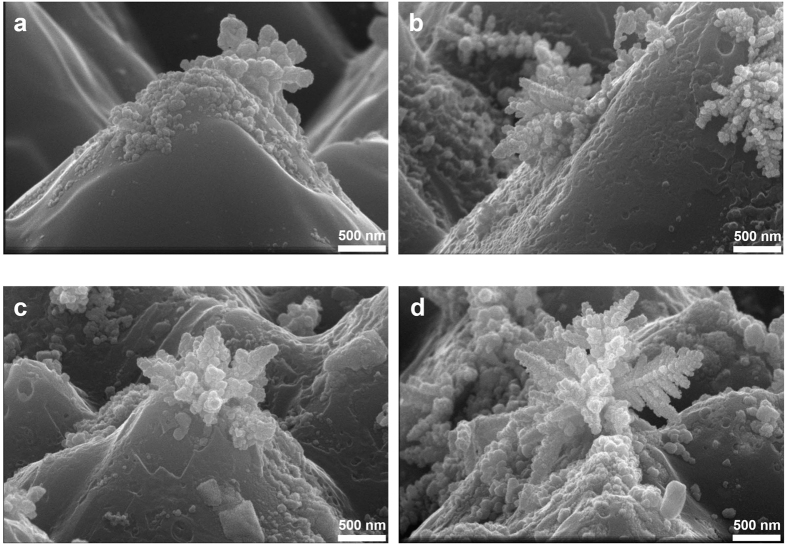
Cross-sectional SEM images of the Ag precipitates that were formed with the injected current densities of (**a**) 2 A/cm^2^, (**b**) 4 A/cm^2^, and (**c**) 5 A/cm^2^ and (**d**) under optimal conditions.

Figure 6a–d show Ag dendrites that are tilted forward by 15° in the cross-sectional images. As shown in Fig. 6a–d, the apexes and slopes of the silicon pyramids have Ag dendrites that are approximately 500 nm or taller. With a higher injected current density, taller Ag dendrites were formed. The amount of electrons injected into the surface of the boron emitter was critical for determining the height of the Ag dendrite. Typically, the glass layer between the boron emitter and the Ag bulk is 500 nm thick^9^; thus, the Ag dendrite that was formed via electron injection ensures direct contact between the boron emitter and the Ag bulk.

**Figure 7:**
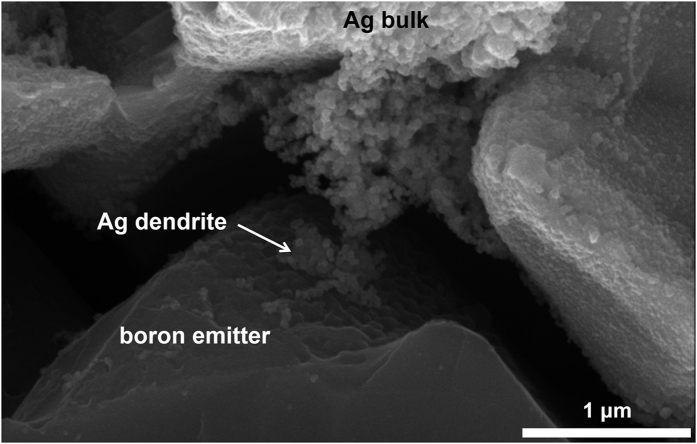
Ag dendrite bridge that was formed between the boron emitter and the Ag bulk.

Figure 7 shows a cross-sectional view of the sample from the boron emitter to the Ag bulk. The sample preparation involved etching of the glass layer using diluted hydrofluoric acid (HF) after the firing process. Figure 7 shows an Ag dendrite that is connected directly from the boron emitter to the Ag bulk. Although the glass layer blocks the current conduction from the emitter to Ag bulk, the Ag dendrite acts as a bridge between the emitter and the Ag bulk, which lowers the contact resistance in the solar cells considerably.

**Figure 8:**
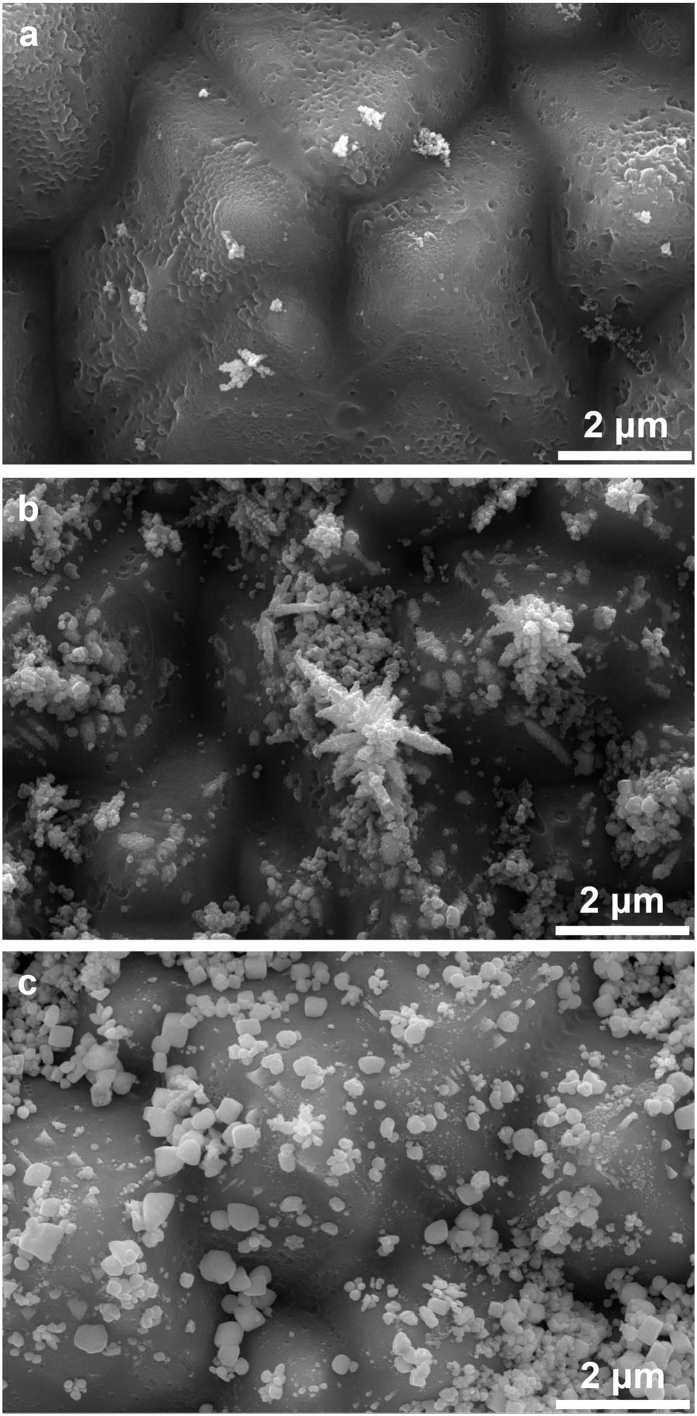
Plane-view SEM images of the Ag precipitates that were fabricated (**a**) without current injection, (**b**) with current injection during the entire (i.e., heating and cooling) firing process, and (**c**) only during heating.

Subsequently, we developed a method to control the injected electrons during the firing process. We performed the same manufacturing process before the firing process and then split the samples into three different conditions by adjusting the time of current injection during the firing process. No current was injected into the first sample, but current was injected into the second and third samples during the entire (i.e., heating and cooling) firing process and during the heating, respectively. In the first sample, nano-sized Ag particles formed after the firing process, as shown in Fig. 8a, which led to a high contact resistance (>100 mΩ∙cm^2^). The second sample exhibited many Ag dendrites and crystallites, as shown in Fig. 8b. The contact resistance of the sample was 0.021 mΩ∙cm^2^, which is more than 10,000 times lower than that of the first sample. In the last sample, Ag formed as crystallites or particles, and no dendrite structure was observed, as shown in Fig. 8c; in this sample, the contact resistance was 22.2 mΩ∙cm^2^. Ag dendrites formed only in the second sample and may have formed during the cooling of current injection via electrochemical migration (ECM) when there was a conducting medium between the biased conductors. During the heating, the Ag was dissolved in the oxygen gas that was present in the ambient air^8^; thus, Ag ions (Ag^+^) were dissolved into the glass layer via oxygen. Positively charged Ag ions moved toward the boron emitter, which was connected to the cathode. The Ag ions crowded near the emitter in the glass layer during the heating of electron injection.

During the cooling, Ag precipitated due to supersaturation over the solubility limit. When an electric potential was applied, as in the second sample, Ag primarily grew as a dendrite. In the absence of any electric potential, Ag grew as a particle and crystallite. The injected electrons during the firing process played a critical role during dendrite formation and markedly reduced the contact resistance. Therefore, Ag dendrites are more important than particles and crystallite for reducing contact resistance.

**Figure 9:**
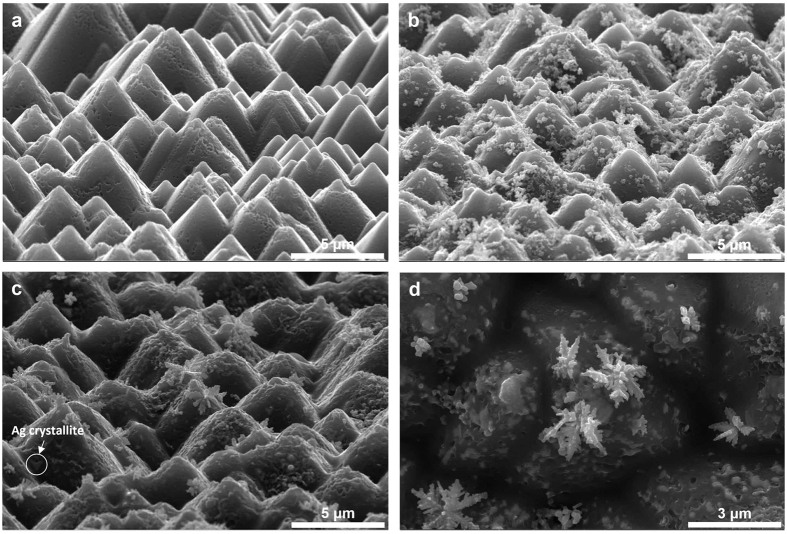
Cross-sectional images that were tilted 15° forward after etching of the Ag bulk and glass layer. The samples were fired under the ambient conditions of (**a**) 100% nitrogen, (**b**) air (nitrogen + 21% oxygen), and (**c**) 100% oxygen. (**d**) Plane-view image of sample (**c**).

We varied the firing atmosphere using different concentrations of nitrogen and oxygen gases. Figure 9a–c show cross-sectional images that are tilted 15° forward after etching of the Ag bulk and the glass layer, and Fig. 9d shows a plane-view image of 9c. In Fig. 9a–c, the samples were fired under 100% nitrogen, air (nitrogen + 21% oxygen), and 100% oxygen, respectively; the image in Fig. 9d also shows the sample that was fired under 100% oxygen. Firing without any oxygen (Fig. 9a) resulted in no formation of Ag particles or dendrites, although the silicon emitter was etched at the apexes and slopes. Firing under 100% oxygen (Fig. 9c) generated more Ag crystallites than firing under air (Fig. 9b); this is clearly visible in Fig. 9d. Under 100% oxygen, higher concentrations of Ag and oxygen ions were dissolved in the glass layer^11^. Then, the etching of silicon accelerated due to the larger amounts of dissolved Ag and oxygen, and Ag nucleated in the region of the etched silicon during the cooling. The nucleated Ag then grew into crystallite formations. Firing under air resulted in lower concentrations of dissolved ions in the glass layer than those obtained under 100% oxygen. Therefore, the silicon was etched less, and fewer Ag nucleation sites were created. During the cooling, supersaturated Ag precipitated in the glass layer or on the silicon surface, which is called Ag particles.

## Discussion

The contact resistance was found to decrease at higher injected current densities due to Ag dendrite formation. Additional and larger Ag dendrites were formed when the injected current density was increased. When the peak wafer temperature was higher, the contact resistance was lowered significantly for low injected current density. For a higher peak wafer temperature, more Ag and oxygen are dissolved in the glass, which are the sources of crystallization; additional Ag particles, crystallites and dendrites can thus be formed. Thus, the peak wafer temperature is an important parameter when attempting to enhance the electrical properties of these systems. The magnitude of the injected current density (i.e., the quantity of electrons injected into the boron emitter) is also significant. If more than a given number of electrons are contained in the emitter, the ECM process occurs during the firing process. The electrical conduction quality can be controlled by injecting electrons into the emitter. ECM is the dissolution and movement of metal ions in the presence of an electric potential, which results in the growth of dendritic structures between the anode and cathode^12^,^13^,^14^. ECM has become increasingly important in the performance and reliability of packaging systems that incorporate electrical contacts^12^. This phenomenon can produce failures that are caused by shorting across closely spaced metallic conductors.

Shorting may also occur in electron-injected, conventional Ag screen-printed solar cell structures. The Ag bulk, glass layer, and silicon emitter act as the anode, transport medium, and cathode, respectively. Therefore, we expected that Ag ECM could occur during the firing process in screen-printed n-type silicon solar cells. Ag dendrites formed at the cathode bridge between the emitter and the Ag bulk; thus, the contact resistance between the boron emitter and Ag bulk decreased to as low as 0.021 mΩ∙cm^2^. This value ensures that no degradation in the fill factor that is caused by contact errors.

During the heating of the firing process, the Ag dissolved in the glass layer by reacting with oxygen gas, as described in equation (1):


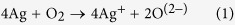


Then, Ag ions moved toward the boron emitter that was connected to the cathode. In the cooling, supersaturated Ag in the glass layer precipitated near the boron emitter and grew as dendrites when an electric potential was applied. In the absence of any electric potential, the Ag grew as particles or crystallites. These results agree with the observations of Hong, K. *et al.*^8^ when an electric potential was present.

**Figure 10:**
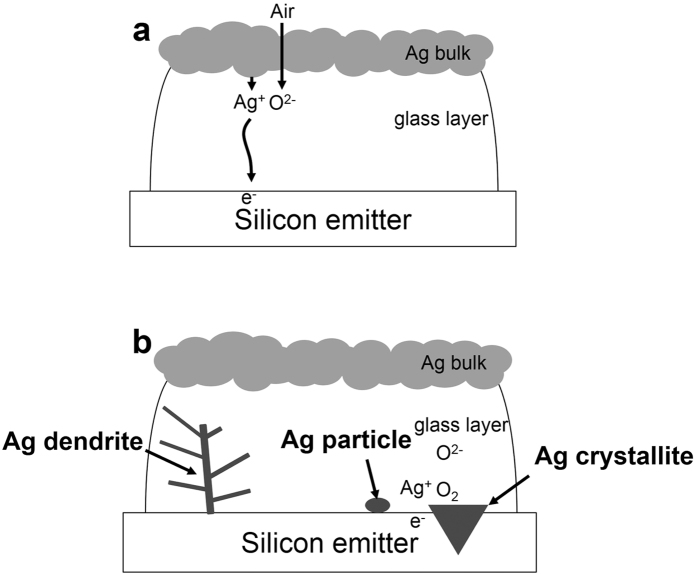
Schematic design of the Ag formation during (**a**) heating and (**b**) cooling.

The above cases are summarized in Fig. 10. The glass layer is strongly expanded to describe the phenomenon in detail. During heating, as shown in Fig. 10a, Ag was from the Ag bulk, and oxygen was from the ambient gases that were dissolved in the glass layer. Then, Ag ions easily moved toward the silicon emitter as if the surface of the silicon emitter was negatively charged. During cooling, as shown in Fig. 10b, Ag ions in the form of dendrites reacted with the electrons that were exposed during the connection to the cathode (i.e., the silicon surface). Also, Ag precipitated in the form of particles due to the small amount of oxygen in the atmosphere. Alternatively, Ag nucleated at the silicon surface and grew in the form of crystallites due to the large amount of oxygen that was dissolved in the glass layer. Ag crystallite formation resulted from the reaction of the Ag ions with oxygen ions and silicon. Another reaction occurred between silicon and oxygen, resulting in silicon etching in the form of silicon dioxide (SiO_2_). In the etched region, Ag nucleated and grew in the form of crystallites. In summary, Ag grew as dendrite formations due to the ECM process and formed two different shapes: particles or crystallites.

## Additional Information

**How to cite this article**: Kim, C. *et al.* Effects of Current-injection Firing with Ag Paste in a Boron Emitter. *Sci. Rep.* **6**, 21553; doi: 10.1038/srep21553 (2016).

## Figures and Tables

**Table 1:** Contact resistances between the Ag bulk and the boron emitter as a function of the injected current density at a peak wafer temperature of 790 °C.

J_injected_ (A/cm^2^)	0	0.5	1.0	1.5	2.0	3.0	4.0	5.0	Lowest result
ρ_c_ (mΩ∙cm^2^)	371	192	111	85.3	75.6	25.2	10.5	10.2	0.021

**Table 2:** Contact resistances between the Ag bulk and the boron emitter as a function of the injected current density at a peak wafer temperature of 830 °C.

J_injected_ (A/cm^2^)	0	2.0	4.0	5.0
ρ_c_ (mΩ∙cm^2^)	32.2	29.9	9.59	0.373
